# The heat is on: reduced detection of floral scents after heatwaves in bumblebees

**DOI:** 10.1098/rspb.2024.0352

**Published:** 2024-08-28

**Authors:** Sabine S. Nooten, Hanno Korten, Thomas Schmitt, Zsolt Kárpáti

**Affiliations:** ^1^ Animal Ecology and Tropical Biology, University of Würzburg, Würzburg, Germany; ^2^ Department of Chemical Ecology, Plant Protection Institute, Centre of Agricultural Research, HUN-REN, Budapest, Hungary

**Keywords:** flower volatiles, VOCs, electroantennography, climate change, *Bombus terrestris*, *Bombus pascuorum*

## Abstract

Global climate change disrupts key ecological processes and biotic interactions. The recent increase in heatwave frequency and severity prompts the evaluation of physiological processes that ensure the maintenance of vital ecosystem services such as pollination. We used experimental heatwaves to determine how high temperatures affect the bumblebees’ ability to detect floral scents. Heatwaves induced strong reductions in antennal responses to floral scents in both tested bumblebee species (*Bombus terrestris* and *Bombus pascuorum*). These reductions were generally stronger in workers than in males. Bumblebees showed no consistent pattern of recovery 24 h after heat events. Our results suggest that the projected increased frequency and severity of heatwaves may jeopardize bumblebee-mediated pollination services by disrupting the chemical communication between plants and pollinators. The reduced chemosensitivity can decrease the bumblebees’ abilities to locate food sources and lead to declines in colonies and populations.

## 1. Introduction

Insect pollination is an essential ecosystem service of global importance and generates considerable economic [1–3] and biodiversity value [4–6]. Yet, this service is in peril as pollinators are in rapid decline at a global scale owing to numerous human-induced disturbances [6,7]. Wide-ranging habitat loss and homogenization, as well as pesticides and pathogens, are major threats to native insect pollinators [7,8]. Climate change and the increase of extreme weather events like heatwaves further exacerbate these negative impacts [9]. This is particularly unsettling, as heatwaves have already become more frequent in recent decades and are projected to increase further in frequency and intensity [10,11]. Reduced flower visitation rates by pollinating insects under heat stress may lead to large reductions in plant reproduction [9].

The interactions between plants and pollinators are mediated by visual cues, like size, shape and colour of the flowers and by olfactory cues, the release of plant volatile organic compounds (VOCs)—the floral volatiles [12–16]. VOCs dissipate from the source by forming patchy distributions of odour filaments with high concentrations interspersed with clean air [17]. Odour-tracking insects fly upwind along the odour plumes to locate their source [18]. Pollinators use a plethora of floral scents as chemical cues to locate food resources at large (habitat size) and small (patch size) scales and to evaluate the condition of the flower [15,19]. However, climate change may jeopardize this chemical communication between plants and pollinators. On the plant side, climatic parameters—ozone and CO_2_ levels, light and temperature—modify the phenology and physiology of the plants and affect VOC production and release, leading to alterations in their ecological functions [20]. For instance, elevated concentrations of ozone and carbon dioxide, recognized as air pollutants, have the potential to significantly degrade floral volatiles and alter the chemical composition of the emitted floral scents [21,22]. Nitrogen oxides and ozone can also disrupt the chemical communication between plants and pollinators by rapidly degrading the compound structure [23,24]. Climatic factors, particularly higher temperatures influence VOC emissions in multifaceted ways, by increasing or decreasing rates, changes in ratios among the scent compounds and unpredictable trends leading to altered attraction by the pollinator [25–29]. On the pollinator side, ozone-rich environments affect their ability to detect floral scents [30,31] and to locate flower volatile resources [32], which can indirectly affect the fitness and survival rate of colonies and populations. Many disruptions in chemical communications are species and compound specific [30,32], and the overall picture remains far from clear.

This study focuses on bumblebees, as they are key pollinators in a wide range of ecosystems [33,34]. Bumblebees are exceptionally well adapted to cold environments [34,35], which makes them particularly vulnerable to ongoing climate warming and heatwaves [36–40]. Stark declines in species and populations have already been reported in areas where opportunities for range shifts are limited [41–43]. Heat stress resistance is lower in mountain and polar populations, and in species with small ranges and declining status trend [44–46]. Flight performance and foraging success are also negatively impacted by high temperatures and simulated heatwaves [47,48]. We investigate whether another key physiological process, the ability to detect floral scents, might be impaired by high temperatures (heatwaves).

Owing to their eusocial strategies, foraging duties are performed by workers, while males are produced for the sexual propagation of the colonies. Nevertheless, males also visit flowers to forage and pollinate during this time [49]. In male bumblebees, selection pressure for heat tolerance might be particularly pronounced, as they spend more time outside of the nest during their lifetime [50]. We simulate heatwaves under laboratory conditions to investigate the bumblebees’ ability to detect floral scents using electroantennography (EAG) recordings. We pose the following questions: (i) do workers and males detect floral scents equally? (ii) Do heat events affect antennal responses? And does this differ between sexes and species? (iii) Can the antennal response of bumblebees recover after heatwaves? Based on previous studies [46–48], we expect that heatwaves will negatively affect antennal responses.

## 2. Methods

### (a) Study species

We selected two species of bumblebees for this study, the common carder bee *Bombus pascuorum* Scopoli 1763 and the buff-tailed bumblebee *Bombus terrestris* Linnaeus 1758. These were selected because they are common and widespread across a wide range of ecosystems in Europe [42] and because the majority of ecosystem services are provided by common and abundant species in the communities [51,52].

### (b) Colony rearing and keeping

*Bombus pascuorum* colonies (*n* = 30) were reared from wild-caught queens emerging from hibernation in the area around Würzburg, Germany (latitude: 49.7833; longitude: 9.9333). The climate is temperate and categorized as Cfb (Marine West Coast Climate) by the Köppen-Geiger classification. Colonies were reared in a climate chamber at 25–27°C, 24 h dark and 50–70% relative air humidity (RH) following the protocol by Ptáček *et al*. [53]. One *B. terrestris* colony was obtained from Behr (Kampen, Germany) and kept in climate chambers at 25°C, 50% RH and a 12 h light/dark cycle. The bumblebees had access ad libitum to bee-collected multi-floral pollen and API-invert, a mix of glucose, fructose and sucrose (Südzucker AG, Mannheim, Germany).

### (c) Climate chamber conditions and heat treatments

**Figure 1.**
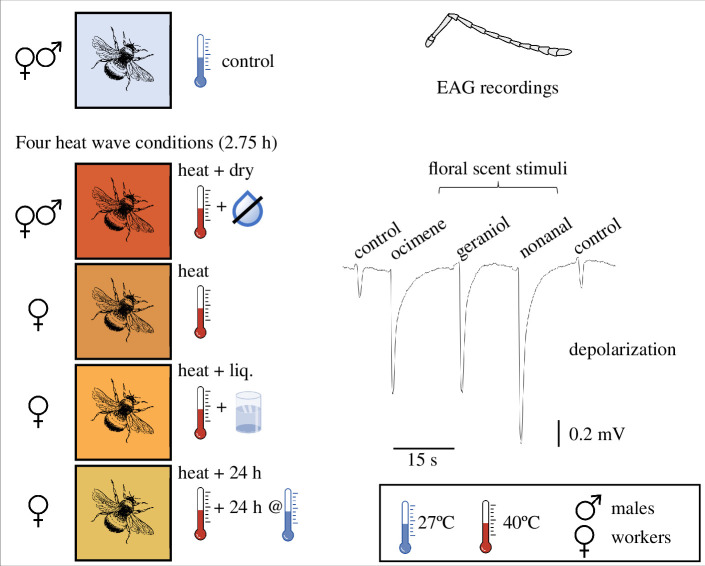
Simulated heatwaves to assess the bumblebees’ ability to detect floral scents. Bumblebees were individually subjected for 2.75 h to experimental heatwaves before their electroantennograms (EAGs) were recorded. The temperature of the four heatwave conditions was 40°C, but they differed by having dry air during the heatwave (heat + dry), allowing access to sugar solution (heat + liq.) or 24 h recovery time at 27°C before the EAGs (heat + 24 h). Control bumblebees were kept at 27°C and 70% air humidity for 2.75 h. The example EAG recording (*B. pascuorum* worker, control) shows the antennal response to control (*n*-hexane) and the three floral scents at the highest concentration (100 μg) (see Methods for detailed description).

One week before commencing the experiments, the bumblebee colonies were kept in a climate chamber at 25–27°C, 50–70% RH, with a 12 h day/night light cycle. To simulate the effects of severe weather events—like heatwaves and droughts—individual bumblebees were removed from the colonies and subjected to four different heat treatments at 40°C for 2.75 h (figure 1). Heat treatment 1 (named hereafter ‘heat’) was conducted with high RH (90%, s.e. ± 2.2; measured with Hygrochron iButtons, Maxim Integrated Products San Jose, USA), treatment 2 (‘heat + liq.’) with high RH and additional access to API-invert ad libitum, treatment 3 (‘heat + 24 h’) with high RH and 24 h resting time at control conditions before testing including API-invert access ad libitum and treatment 4 (‘heat + dry’) with dry air (15%, s.e. ± 6.6). The four different treatments were selected to simulate the effects of heatwaves (treatment 1) and heatwaves with low relative air humidity (4), and to assess whether bumblebees can ‘recover’ from heat events in 24 h (3) and whether effects are due to the bumblebees lack of energy and water loss [54] during the heat events (2).

For each heat treatment, a bumblebee was placed in a 50 ml Falcon tube (Sarstedt, Nümbrecht, Germany) on a foam inlay. For the ‘dry’ heat treatment, 10 g of silica gel orange (Roth, Karlsruhe, Germany) was added below the foam. The lids were loosely placed on top to ensure sufficient airflow. All heat treatments were conducted in a climate cabinet (model 100–800, Memmert, Schwabach, Germany). As a control, bumblebees were placed individually in 50 ml Falcon tubes and placed in the climate chamber at 25–27°C and 50–70% relative air humidity for 2.75 h (control).

The heat treatments were conducted in 2022. *B. pascuorum* workers and males were subjected to these experiments in the first half of July (colony rearing started in April) and *B. terrestris* in mid-December. Treatments were alternated randomly within each species.

The silica gel orange was tested for any potential scents that could interfere with the three flower volatiles presented to the bumblebees. To collect the headspace volatiles of dry silica orange gel, we used the solid phase microextraction (SPME) technique. Dry silica gel(37 g) was placed into a 100 ml Erlenmeyer flask covered with aluminium foil. The SPME fibre (CAR/PDMS, 0.53 mm, Supelco, Sigma-Aldrich, Bellefonte, PA, USA) was inserted into the Erlenmeyer flask headspace for 20 min at 40°C. After volatile collection, the fibre was inserted for 1 min into the injection port (250°C) for thermal desorption of the sample for the gas chromatography-mass spectrometry (GC-MS) analysis (Agilent 7890B GC and 5977 MS, Agilent Technologies, Waldbronn, Germany). The GC was equipped with a HP−5MS UI capillary column (30 m × 0.25 mm × 0.25 μm, J & W Scientific, Folsom, CA, USA). Helium was used as a carrier gas with a constant flow of 1 ml/min. The oven temperature was initially held at 40°C for 1 min, then raised to 150°C at 5°C/min and 300°C at 50°C/min where it was held for 5 min. The MS was operated in electron impact (EI) ionization mode at 70 eV, scanning *m/z* 40–600, at 2.6 scans/s. Before each measurement, fibres were conditioned at 250°C in the split/split-less injection port of the GC-MS for 2 min. There was no scent emitted from the silica orange gel. However, we found siloxane contamination either from the silica gel or from the GC column (see GC-MS chromatogram in electronic supplementary material, figure S1).

### (d) Preparation of flower volatiles/chemical compounds

We chose three synthetic flower volatiles from three major compound classes that are common in many flowering plants and either show attractive behaviour or elicit antennal responses to a wide range of generalist pollinators, including bumblebees [55–61]. The monoterpene ß-ocimene as a racemic mixture of *cis* and *trans* (CAS: 13877-91-3, Sigma-Aldrich, mixture of isomers, purity ≥90%), named ‘ocimene’ hereafter, the monoterpene geraniol (CAS: 106-24-1, Sigma-Aldrich, purity: 98%) and the aldehyde nonanal (CAS: 124-19-6, Sigma-Aldrich, purity ≥98%). These compounds were dissolved in *n*-hexane (CAS: 110-54-3, Merck KGaA, for gas chromatography MS SupraSolv), and 10 μl of the corresponding dilutions (0.1, 1 and 10 μg/μl) of the compounds were deposited on a filter paper (1 × 1 cm), which was then placed into a Pasteur pipette and immediately used as a stimulus cartridge in electroantennography (EAG) experiments.

### (e) Physiological experiments—EAG

To assess what extent the heat treatments decrease the bumblebees’ ability to detect floral resources, we performed EAG recordings with the three flower VOCs. The EAG recordings measure the depolarization amplitude of all active olfactory sensory neurons in response to a stimulus [62]. For the recordings, the antenna of the insect was excised and inserted into a glass capillary (ID 1.17 mm, Syntech, Kirchzarten, Germany) filled with Ringer solution [63] and attached to the reference silver electrode. Half of the last (distal) segment of the antenna was cut and inserted into the recording glass electrode, which was also filled with Ringer solution. The antennal signal was 10 times pre-amplified, converted to a digital signal using a DC amplifier interface (IDAC-2, Syntech) and recorded with GC-EAD software (GC-EAD 2014, v. 1.2.5, Syntech). Antennae were stimulated for 0.5 s using a Stimulus Controller (CS-55, Syntech). The stimulation air (2 l/min) was led into a constant, charcoal filtered and humidified air stream (2 l/min). The solvent of the flower volatiles (*n-*hexane) was used as a control stimulus. The sequence of each antenna stimulation was as follows (see also figure 1): *n-*hexane—the three flower volatile compounds at low (1 μg) concentration. This sequence was repeated for mid (10 μg) and high (100 μg) concentrations. The order of the three flower volatiles was randomized within each concentration and for each tested antenna.

We tested a total of 190 bumblebees. Of these, 106 were *B. pascuorum* workers (20 control, 22 heat, 23 heat + liq., 19 heat + 24 h and 22 heat + dry), and 14 males (7 control and 7 heat + dry). There were 50 *B*. *terrestris* workers (10 in the control and each of the four treatments) and 20 males (10 in the control and 10 in heat + dry). All bumblebees were tested subsequent to the heat treatments, except for the heat + 24 h where bees were tested after 24 h.

### (f) Analyses

All statistical analyses were carried out using the software *R* v. 4.2.0 [64]. We used generalized linear mixed modelling (glmmTMB) in the package *glmmTMB* [65] to investigate the effects of heat treatments on the bumblebees’ antennal response to the three floral volatiles at three different concentrations. EAG antennal responses (amplitude) were adjusted by subtracting from the accompanying control stimulus (*n*-hexane) in each session to compensate for solvent and mechanoreceptive responses [66,67]. We used the function ‘glmmTMB, which allows the fitting of mixed effect models with random and fixed effects, for zero-inflated data; a zi-gamma distribution and log link were used to fit the right-skewed distribution of the data [68]. The functions ‘emmeans’ and ‘contrast’ with Tukey’s HSD in *emmeans* were used for *post hoc* comparisons [69]. (i) To assess whether workers and males detect floral scents equally, we used responses of bumblebees that did not undergo heat treatments (= control), fitted a model with random (= nest identity and individual) and fixed effects (= sex, species and concentration) and used *post hoc* comparisons in *emmeans* to test for differences between sexes and species *within* each of the three concentrations. We also tested for a ‘dose response’ by comparing responses between concentrations. (ii) and (iii) To analyse the effects of heat events on antennal responses, we fitted the same model as described above with treatment and concentration as fixed effects, nest identity and individual as random effects, and used *post hoc* comparisons for differences between control and the heat treatments within each species, sex and concentration (control versus heat + dry for males, and control versus heat, heat + liq., heat + 24 h and heat + dry).

We additionally used standardized effect statistics to quantify the magnitude of the experimental effects [70]. To this end, we calculated standardized effect sizes (Cohen’s *d*) for each heat treatment versus control group [71]; Cohen’s *d* was calculated by subtracting heat treatment group means from control group means and dividing by the square root of the pooled standard deviations. Negative effect size values indicate a decreased antennal response to flower volatiles after the heat treatment and positive ones the opposite. Following Cohen [71], we consider effect size values <0.2 as small, >0.5 as medium and >0.8 as large.

## 3. Results

### (a) Do workers and males detect floral scents equally?

**Figure 2.**
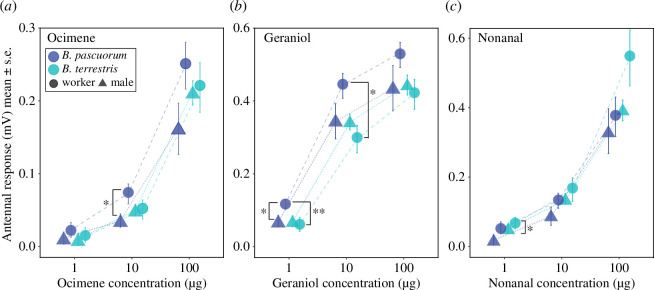
Antennal responses of worker and male bumblebees to three floral volatiles at three concentrations. Panels (*a*)–(*c*) show responses of workers (circles) and males (triangles) from two bumblebee species (*B. pascuorum* and *B. terrestris*, at control conditions) to (*a*) ocimene, (*b*) geraniol and (*c*) nonanal. Note *y*-axis scale differs for ocimene. Asterisks indicate statistical significance for comparisons between sexes and species within volatile dose (*p < 0.05; **p < 0.01).

All three compounds resulted in concentration-dependent antennal responses in both species and sexes (figure 2; *p *< 0.0001; electronic supplementary material, tables S1–S3). Responses were on average lowest at 1 μg and increased by up to one order of magnitude at the 100 μg dose (figure 2). Geraniol and nonanal elicited higher responses than ocimene (on average twofold). Overall, there was no significant difference between species. However, pairwise comparisons showed that antennal responses of *B. pascuorum* workers were significantly stronger (by 1.5-fold) than *B. terrestris* workers to geraniol at low (*p* = 0.007) and medium doses (*p* = 0.025; electronic supplementary material, table S2). Antennal responses were only significant between sexes for nonanal (*p* = 0.043; electronic supplementary material, table S3), with *B. terrestris* workers responding on average twofold stronger than males at low doses (*p* = 0.039). Pairwise tests also showed significant differences between sexes for *B. pascuorum* at medium dose ocimene (*p* = 0.047) and *B. terrestris* at low dose geraniol (*p* = 0.032).

### (b) Do heat events affect antennal responses?

**Figure 3.**
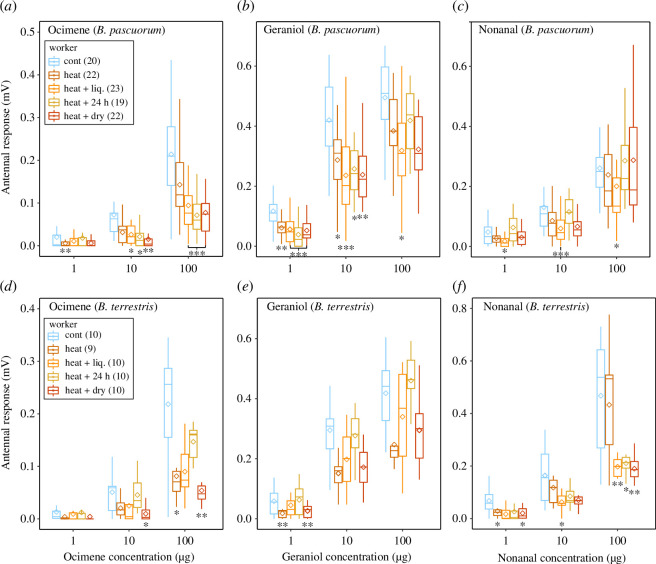
Antennal responses of bumblebee workers to three floral volatiles after control conditions and four experimental heatwaves. Boxplots show the mean (diamond shape), median, upper and lower quartile. Upper panels show responses of *B. pascuorum* (*a,b,c*) and lower ones *B. terrestris* (*d,e,f*) to ocimene (left panels), geraniol (middle) and nonanal (right) at three concentrations. Note *x*-axis scale differs. Asterisks indicate statistical significance for comparisons between control and experimental heatwaves within volatile dose and sexes (*p < 0.05, **p < 0.01 and ***p < 0.001). Numbers in brackets show sample size.

The heat treatments significantly decreased antennal response to the three floral scents in workers of both species (*p* < 0.0001; figure 3; electronic supplementary material, tables S4 and S5). Antennal responses were also dose dependent (*p* < 0.0001) and increased with concentration. *B. pascuorum* workers showed an interaction effect of dose and treatment for ocimene where treatment effects increased with dose (*p* < 0.0001; electronic supplementary material, table S4). Antennal responses in *B. pascuorum* workers were significantly reduced (on average by 27–79%) in 18 of 36 instances (figure 3; electronic supplementary material, table S4). Standardized effect sizes show that reductions increase from heat to heat + dry for ocimene (figure 5*a*) and are less pronounced in heat + 24 h for nonanal (figure 5*e*). There is little consistent pattern among heat treatments in terms of severity, i.e. heat + 24 h does not have a consistently less severe effect and heat + dry does not have a consistent effect of being most severe, except for ocimene where heat + dry effect increased in severity with concentration (figure 5). In *B. terrestris* workers, significant reductions (by 42–81% on average) occurred in 12 of 36 instances (figure 3; electronic supplementary material, table S5). Reductions increase with concentration (as shown by the size of standardized effect sizes) for ocimene (figure 5*b*) and nonanal (figure 5*f*) and are significant at high concentrations (figure 3*d*,*f*). Heat + 24 h treatments show a ‘recovery effect’ for ocimene and geraniol, as antennal responses are comparable with the control (figure 3*d*,*e* and figure 5*b,d*). The effect of heat + dry is consistently most severe for ocimene and nonanal (figure 3*d*,*f* and figure 5*b,f*).

**Figure 4.**
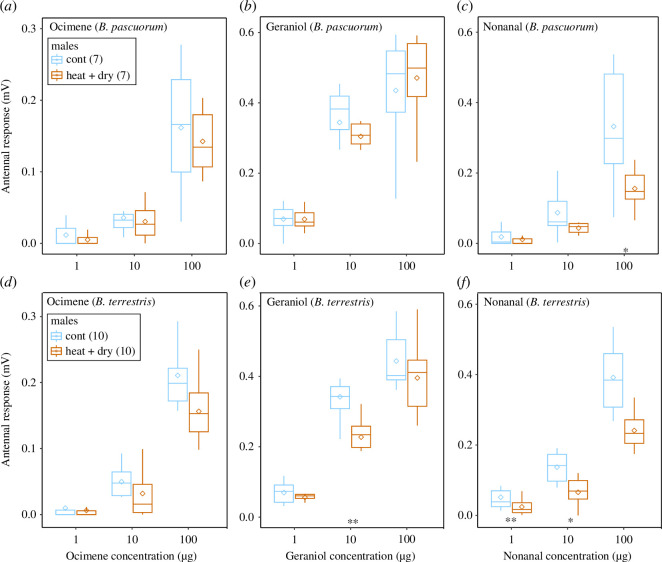
Antennal responses of bumblebee males to three floral volatiles after control and experimental heat + dry conditions. Boxplots show the mean (diamond shape), median, upper and lower quartile. Upper panels show responses of *B. pascuorum* (*a,b,c*) and lower ones *B. terrestris* (*d,e,f*) to ocimene (left panels), geraniol (middle) and nonanal (right) at three concentrations. Note *x*-axis scale differs. Asterisks indicate statistical significance for comparisons between control and experimental heatwaves within volatile dose and sexes (*p < 0.05, **p < 0.01 and ***p < 0.001). Numbers in brackets show sample size.

**Figure 5.**
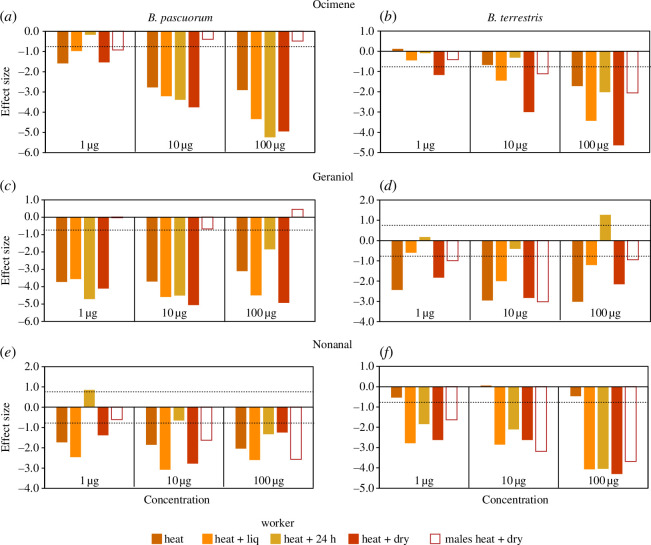
Effect sizes of antennal response of bumblebee workers and males to three floral volatiles. Graphs show the four heat treatments (heat, heat + liq., heat + 24 h and heat + dry) of workers in comparison to control, and one heat treatment of males (heat + dry) to control (open bars). Negative effect size values indicate decreased antennal responses after heat treatments than the control, positive values the opposite. Panels (*a*), (*c*) and (*e*) show responses of *B. pascuorum* and panels (*b*), (*d*) and (*f*) show responses of *B. terrestris* to ocimene (upper panel), geraniol (middle) and nonanal (lower). Dashed lines (+0.8 and −0.8) indicate large effect sizes. Note *y*-axis scales differ.

Overall, antennal responses of male bumblebees significantly decreased in *B. terrestris* for geraniol and nonanal (*p* = 0.009 and *p *< 0.0001; figures 4 and 5; electronic supplementary material, table S6). Responses increased significantly with concentration for both species (*p *< 0.0001). Significant reductions after heat + dry treatment ranged on average from 26% to 53% (figure 4; electronic supplementary material, table S6). They occurred in *B. terrestris* to geraniol at medium and nonanal at low and medium concentrations (figure 4*e,f*). *B. pascuorum* males seem to be more robust (only one significant reduction to nonanal at high concentration, figure 4*c*) than *B. terrestris* males (figure 4). Effect sizes show that reductions in antennal responses after heat + dry treatments were more pronounced in workers than in males (figure 5), for ocimene (both species, figure 5*a,b*) and for geraniol (*B. pascuorum*, figure 5*c*).

## 4. Discussion

Ongoing climate change disrupts key ecological processes and biotic interactions. This prompts the evaluation of physiological processes that ensure the maintenance of vital ecosystem services such as pollination. This study used simulated heatwaves to assess how high temperatures affect the bumblebees’ ability to detect floral scents. We found that simulated heatwaves led to a strong reduction in antennal responses to floral scents in both tested bumblebee species. Reductions were more pronounced in workers than in males. Bumblebees showed no consistent pattern of recovery 24 h after the heat events. Our results indicate that heatwaves may compromise bumblebee-mediated pollination services by disrupting the chemical communication between plants and their pollinators.

### (a) Simulated heatwaves reduce antennal responses in bumblebees

The heat events led to a reduced antennal response to the floral scents in both bumblebee species and sexes. Antennal responses were severely diminished in workers, where chemosensitivity was reduced by up to 80%. Responses decreased the most at middle and high doses. Reductions were particularly pronounced for ocimene and least for geraniol. Thus, our study shows that heat stress might affect odour detection at the physiological level, leading to diminished foraging performances of pollinators [48]. This impaired odour detection could hamper the long-range attraction and small-scale patch orientation of foragers, and result in compromised pollination services with the increasing frequency and severity of heatwaves. Under natural conditions, bumblebees might be able to counteract some of these effects by behavioural strategies of thermoregulation, such as seeking out thermal refuges and adjusting their temporal activity pattern [40]. However, persistent heatwaves over a larger period can still threaten bumblebee populations, as their flight performance is diminished at high temperatures [47], and successful foraging bouts are reduced [48].

Heat stress can cause metabolic and morphological damage to insects. Increased temperatures are known to damage the morphological structure of the olfactory sensilla on the antennae by disorganizing and scattering the olfactory sensilla and hinder the transmission of odorant molecules to the olfactory receptors [72,73]. Heat events trigger the expression of heat shock proteins (HSPs), which help denatured proteins to refold and regain function [74]. The HSP expression in bumblebees is highly conserved regardless of population origin [75] but patterns differ among tissues [76]. High temperatures alter the gene expression in antennae of *Drosophila* by downregulating olfactory and upregulating HSP genes [77]. This could also be expected to occur in bumblebees. Odour detection involves a range of proteins, including odorant receptors [78], odorant binding [79,80] and odorant degrading proteins [81]. HSPs might protect these proteins during heat events to a certain degree but some may nevertheless denaturate. HSP protection is also costly as it uses ATP [74], and heat-stressed bumblebees might have less expendable energy for non-essential metabolic functions (e.g. odour detection).

### (b) Wild-sourced species appear to be less resilient to heat events than commercial ones

Workers of the wild-sourced species, *B. pascuorum,* were less resilient than those of the commercially obtained *B. terrestris*. The size of the reduction in antennal responses of *B. pascuorum* was larger and significant in more instances. One might speculate that wild bumblebees might be more sensitive to environmental stress, such as heatwaves, than commercial ones, which is also exemplified by their commercially induced range expansion [82]. Alternatively, the two species might differ in their sensitivity. Further comparative research with more species could shed light on these trends.

### (c) Bumblebees’ antennal response show no consistent pattern of ‘recovery’ after heat events

Bumblebees showed no consistent pattern of ‘recovery’, when tested 24 h after heat events. The antennal response after a period of 24 h resting time at ambient temperature and humidity was comparable with the control for *B. terrestris* to ocimene and geraniol and for *B. pascuorum* to nonanal. In all other instances, the antennal responses to the floral volatiles were similarly diminished as those tested directly after the heat event. HSP gene expression levels decreased after the 24 h recovery period following heat shock [83]. This suggests that bumblebees may exhibit a differential expression pattern of HSPs, which could explain the varying recovery results. While HSPs are present in *B. terrestris* [76], and most likely also in *B. pascuorum*, it is currently unclear whether they play a role in protecting the antennae from heat stress. Further research is needed to confirm this hypothesis.

### (d) Floral scent detection is comparable in workers and males

Overall, antennal responses to the floral scents were slightly stronger in workers than in males, though differences were generally not statistically significant (electronic supplementary material, tables S1–S3). This trend was more pronounced in *B. pascuorum* than in *B. terrestris*. Our result suggests that both sexes can detect the same suite of floral scents. Thus, both sexes might express the same set-up of olfactory genes on the dendrites of olfactory receptor neurons in the sensillum. Remaining sex differences may then be attributed to different expression levels of these olfactory receptor genes [84]. Sex differences in antennal responses are rarely considered in the literature, and results remain inconsistent. Antennal responses to plant volatiles are higher in female hawkmoths [66] and cabbage seed weevils [85]; no sex differences are evident in mining bees [86] and moths [87]. Even within a single genus, responses are not uniform, as antennal responses of males are stronger in *B. terrestris* but equal between the sexes in *B. hypnorum* [88].

### (e) Workers are more susceptible to heat events than males

Males were more heat resilient than workers (figure 5). While the responses of workers (to heat + dry treatment) were reduced by up to 80%, those of males were reduced by up to 50% on average. Male bumblebees can be surprisingly heat tolerant [45], which may be due to differentially expressed HSP patterns, such as those found in Lepidoptera [89]. Also, workers are responsible for locating and gathering food for the colony, which requires highly developed olfaction. It is possible that workers have more olfactory receptors tuned to the volatiles released by flowers, which makes them more sensitive to odour cues [90]. Any damage to the receptors may, therefore, have a greater impact on the workers’ ability to locate food and perform their tasks effectively compared with males.

## 5. Conclusions

Bumblebees perform vital pollination services in managed and native ecosystems. Our results show that heatwaves diminish the bumblebees’ ability to detect floral scents, and this is more pronounced in workers than in males. This reduced olfactory detection of host plant volatiles can jeopardize foraging and host plant location by bumblebees. If bumblebees’ olfactory system does not adjust to environmental changes, colony survival might be in peril leading to far-reaching consequences for populations and species. Future research should focus on investigating heat stress effects on the cellular, morphological and physiological changes of sensilla and receptors, and link these with behavioural experiments and foraging success.

## Data Availability

Data and R code are available in the Dryad repository [91]. Supplementary material is available online [92].
